# Polysulfone/Polyamide-SiO_2_ Composite Membrane with High Permeance for Organic Solvent Nanofiltration

**DOI:** 10.3390/membranes8040089

**Published:** 2018-10-03

**Authors:** Qin Liu, Xing Wu, Kaisong Zhang

**Affiliations:** 1 Key Laboratory of Urban Pollutant Conversion, Institute of Urban Environment, Chinese Academy of Sciences, Xiamen 361021, China; qinliu@iue.ac.cn (Q.L.); xwu@iue.ac.cn (X.W.); 2 University of Chinese Academy of Sciences, Beijing 100049, China

**Keywords:** silica, polysulfone, composite membrane, polyamide

## Abstract

To improve the filtration performance and properties of organic solvent nanofiltration (OSN) membranes, we firstly introduce nanoporous silica (SiO_2_) particles into the polyamide (PA) active layer of polysulfone (PSf) membrane via an interfacial polymerization process. Results from the study revealed that introduction of SiO_2_ influenced the properties of PSf/PA-SiO_2_ composite membranes by changing the surface roughness and hydrophilicity. Moreover, results also indicated that nanoporous SiO_2_ modified membranes showed an improved performance of alcohols solvent permeance. The PSf/PA-SiO_2_ composite membrane modified by 0.025 wt % of SiO_2_ reached a permeance of 3.29 L m^−2^ h^−1^ bar^−1^ for methanol and 0.42 L m^−2^ h^−1^ bar^−1^ for ethanol, which were 20.0% and 13.5% higher than the control PSf membrane (permeance of 2.74 L m^−2^ h^−1^ bar^−1^ for methanol and 0.37 L m^−2^ h^−1^ bar^−1^ for ethanol). Conclusively, we demonstrated that the increase of membrane hydrophilicity and roughness were major factors contributing to the improved alcohols solvent permeance of the membranes.

## 1. Introduction

Organic solvent nanofiltration (OSN) or solvent resistant nanofiltration (SRNF) have attracted major attention as promising technologies in the purification and separation process, especially in lube oil dewaxing [1], enhanced catalysis [2] and isolation and concentration of pharmaceuticals [3]. Compared to conventional separation processes such as distillation, evaporation, adsorption, extraction and chromatography, OSN and SRNF show several advantages including high efficiency, low energy consumption and operational stability [1].

Generally, OSN membranes could be classified as organic polymeric membranes and inorganic ceramic membranes according to the membrane materials applied for membrane fabrication [4,5,6,7]. Common polymers such as polyimide (PI) [8,9,10,11], polybenzimidazole (PBI) [12], polyetherimide (PEI) [13], polydimethylsiloxane (PDMS) [14,15,16], and polyacrylonitrile (PAN) [17,18,19,20] have been mostly developed in membrane fabrication processes. These membranes show good filtration performance and a high possibility of up-scaling. However, due to the high viscosity or large molecular volume of organic solvent, these membranes have relatively low permeance for organic solvent, which greatly limits the industry application of OSN membranes. It is reported that the permeance of polar organic solvents can be enhanced with the improvement of hydrophilicity for OSN membranes [21]. To date, considerable effort has been devoted to improving the hydrophilicity of conventional membranes by employing various techniques, including coating [22,23], blending [9,24,25] and surface grafting polymerization [26]. Among these fabrication methods, blending membranes with inorganic nanoparticles such as Metal-Organic Frameworks (MOFs) [6,27,28], metals [29] or carbide [30,31] has been widely used to fabricate inorganic-organic composite membranes. Among these nanoparticles, silica (SiO_2_) has been proved to be a promising candidate to blend with membrane during the process of casting solution preparation [24,25], and has also shown benefits such as low cost, a simple synthesis process, high chemical stability and excellent hydrophilicity. However, blending silica with OSN membranes during the interfacial polymerization (IP) process is rarely reported [32]. 

As a common organic membrane material, polysulfone (PSf) has been widely used in the membrane fabrication industry. PSf-based nanofiltration membranes show excellent filtration performance, good mechanical strength and durability in water treatment [33]. Recently, researchers began to focus on the application of PSf in the OSN membranes fabrication due to the stability of PSf in alcohol organic solvents [34,35,36,37]. However, to the best of the authors’ knowledge, current PSf-based OSN membranes still show a low permeance of alcohol solvents [34]. Therefore, to improve on the large-scale production of PSf-based OSN membranes in industry, it is critical to improve the properties and filtration performances of OSN membranes.

In this work, we first introduced mesoporous silica into the PSf-based nanofiltration membranes during the interfacial polymerization (IP) process. The filtration performance of the prepared OSN membranes demonstrated that SiO_2_ obviously improved the alcohol solvents permeance of PSf/PA membranes, with a minimal decrease of the rejection performance. To further investigate the property of OSN membranes, a series of analyses, such as SEM, AFM, water contact angle and a long-term filtration test were also carried out to characterize the effect of SiO_2_ nanoparticles on the structure and performance of the OSN membranes.

## 2. Experiment

### 2.1. Materials

Polysulfone was purchased from Solvay S.A., Shanghai, China. 1-methyl-2-pyrrolidinone (NMP, ammonium (25~28% NH_3_·H_2_O), sodium lauryl sulfate (SLS), triethylamine (TEA), hexane, methanol, and ethanol were purchased from Sinopharm Ltd., Shanghai, China. Piperazine (PIP), trimesoyl chloride (TMC) and tetraethylorthosilicate (TEOS) were purchased from Tokyo Chemical Industry Ltd., Hashimoto, Japan. Cetyl trimethyl ammonium bromide (CTAB), rose-bengal (RB), bromothymol blue (BTB), crystal violet (CV), methyl orange (MO) and (±)-Camphor-10-sulfonic acid (CSA) were purchased from Aladdin Industrial Corporation, Shanghai, China. All chemicals used were of analytical grade and were used without further purification. The water used in all experiments was distilled water.

### 2.2. Synthesis of Mesoporous Silica

Mesoporous silica was prepared as described previously [26]. Typically, 2.1 mL of 2M NaOH aqueous solution was added into 288 mL distilled water under mechanical stirring at room temperature. Then, 0.6 g CTAB was introduced into the above mixture solution. After that, the solution was heated at 80 °C until a clear solution was obtained and TEOS (3 mL) was added dropwise with vigorous stirring. The mixture reaction solution was kept stirring at 80 °C for 2 h. After reaction, the product was centrifuged, collected, and further washed with ethanol for several times to completely remove the template (CTAB), and dried in an oven at 50 °C. Finally, the mesoporous silica powder was obtained.

### 2.3. Preparation of PSf Substrates

The PSf substrates were prepared by phase inversion technique according to reported procedures [38]. Typically, the casting solution containing 16.5 wt % PSf, 0.3 wt % DI water, 0.3 wt % SLS and 82.9 wt % NMP was prepared. To fabricate a PSf substrate membrane, the casting solution was cast 200 μm thick onto a polyester nonwoven fabric, and immersed in a fresh DI water bath immediately. The thickness of prepared PSf support was 155 μm and was stored in NaHSO_3_ solution (1 wt %). 

### 2.4. Preparation of PSf/PA-SiO_2_ Nanocomposite Membranes

PSf/PA-SiO_2_ nanocomposite membranes were prepared on the PSf substrate by IP process. Typically, the aqueous phase was prepared by homogeneously mixing 91.3 mL of deionized water with 1.6 g of PIP, 3 g of CSA, 3 g of TEA, 0.1 g SLS, and different amounts (0.00 wt %, 0.0125 wt %, 0.025 wt %, 0.05 wt %, and 0.075 wt % based on the aqueous solution weight) of dry SiO_2_ at room temperature. Then, the aqueous phase solution was casted on the PSf substrate membrane. After keeping for 45 s, the residual solution was removed from the PSf substrate by tissue papers. Then, the organic phase solution formed by dispersing 0.35 g of TMC in 100 mL of hexane solvent, was poured on the above wetted PSf substrate. After 20 s of IP reaction, the membrane was placed in an oven at 60 °C for 2 min for further cross linking. Finally, the membranes were stored in deionized water for further performance evaluation. According to the amounts of SiO_2_ in the aqueous phase, the membranes were labelled as PSf/PA, PSf/PA-SiO_2_ 0.0125%, PSf/PA-SiO_2_ 0.025%, PSf/PA-SiO_2_ 0.05%, PSf/PA-SiO_2_ 0.075%.

### 2.5. Characterization of Membranes

The morphological structures of PSf-SiO_2_ membranes were characterized using a field emission scanning electron microscopy (S-4800, HITACHI Ltd., Tokyo, Japan) conducted with an energy dispersive X-ray (EDX) spectrometer (Oxford 6587, HITACHI Ltd., Tokyo, Japan). The hydrophilicity of the membranes was determined by measuring the water contact angles of the membrane surface with a contact angle goniometer (CMA200, KSV Instruments Ltd., Helsinki, Finland). At least 3 different locations on one membrane sample were measured to obtain an average value of the contact angles in each membrane.

The surface roughness of membranes was determined using a Dimension 3100 atomic force microscopy (AFM) device (Bruker Ltd., Billerica, MA, USA), under ambient condition, with a scanning area of 2 × 2 μm^2^. At least 3 different spots on each membrane sample were recorded to obtain an average value. Moreover, the roughness value of the membranes was expressed as root mean-square roughness (R_q_), average roughness (R_a_) and maximum vertical distance (R_z_) between the highest and lowest point of the membrane surface. The results were showed in the supporting information (Figure S2 and Table S1). 

The filtration performance of the membranes was measured using a dead-end cell (HP4750 Sterlitech Ltd., Washington, DC, USA) at a pressure of 3.5 bar achieved with nitrogen. The effective area of the membrane was 14.6 cm^2^ and 25 mL alcohol solvent of dye at 20 μM was charged in the cell. The solvent was magnetically stirred at 500 rpm. After 1 h filtration, permeate solvent was collected for permeance and rejection measurements.

The solvent permeance was determined according to the following Equation:

(1) $$J=\frac{Q}{A\times \Delta P}$$

where Q is the permeance rate (L h^−1^), *A* is the effective filtration area (m^2^), and ΔP is the pressure difference (bar).

In order to make a comparison between the permeance based on PSf/PA-SiO_2_ membranes intuitively, a normalized permeance was employed in which the results were further calculated with the follow Equation:

(2) $$\mathrm{Normalized}\;\mathrm{permeance}\;\left(\%\right)=\left(\frac{J_{PSf-SiO_{2}}}{J_{PSf}}\right)\times 100$$

where $J_{PSf-SiO_{2}}$ (L m^−2^ h^−1^ bar^−1^) is the solvent permeance of PSf/PA-SiO_2_ membranes, $J_{PSf}$ (L m^−2^ h^−1^ bar^−1^) is the solvent permeance of PSf membrane.

The rejection (*R*) was evaluated using following Equation:

(3) $$R\left(\%\right)=\left(1-\frac{C_{p}}{C_{f}}\right)\times 100$$

where *C_p_* and *C_f_* represent the dye concentrations of permeate and feed solution, respectively. Concentration of RB, BTB, CV, MO in the solvent were analyzed by UV 2450-vis spectrophotometer (Shimadzu, Kyoto, Japan). The solvent permeance and dye rejection were measured by at least three membrane samples and the results were average values of these measurements.

## 3. Results and Discussion

### 3.1. Membrane Characterization

The EDX analysis of the PSf/PA-SiO_2_ membrane and the PSf/PA membrane are given in Table 1. Only PSf/PA-SiO_2_ membrane with 0.075 wt % SiO_2_ and PSf/PA membrane were discussed in this section. As shown in Table 1, a new element Si with atomic ratio 0.88 appeared, while the percentage atomic ratio of O also increased, which confirmed that the SiO_2_ nanoparticles were successfully embedded into the PA layer of PSf substrate.

**Figure 1:**
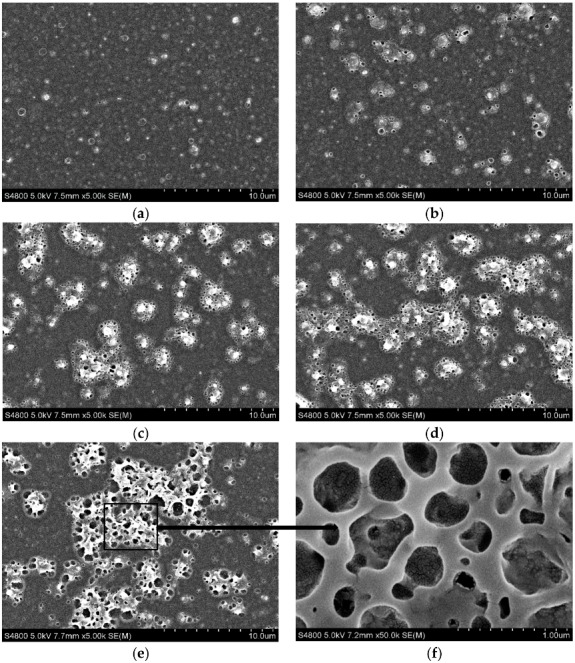
SEM pictures of surface morphology of (**a**) PSf/PA and PSf/PA-SiO_2_ composite membrane with the mass fraction of SiO_2_ of (**b**) 0.0125 wt %; (**c**) 0.025 wt %; (**d**) 0.05 wt %; (**e**) 0.075 wt % in the magnification of 5000 times; (**f**) the specialized area of (**e**) in the magnification of 50,000 times.

SEM images of the PSf/PA membrane with different amounts of SiO_2_ are shown in Figure 1. Compared to the uniform and smooth morphological surface of PSf/PA membrane (Figure 1a), there are some bulges on the surface of SiO_2_ doped membranes due to the embedding of SiO_2_ nanoparticles, which make the surfaces rougher. The amount of bulges increased with the increase of the doped SiO_2_ concentration. A similar mechanism was observed for carbon dots nanoparticles introduced into the PA layer of PSf substrate, as demonstrated in our previous work [31]. Furthermore, after the increased addition of SiO_2_, some showerheads-like structure appeared on the bulges, and the size of the voids in the showerhead-like structure increased with the increased concentration of SiO_2_ nanoparticles. The introduction of the SiO_2_ nanoparticles into PA layer during IP process can influence the formation of PA dense layer considerably, thus allowing the hydrophilic nanoparticles to adsorb unto PIP monomers, which then forms the active centers for the polymerization of PIP and TMC. This interaction makes the surface of the modified membranes different from the unmodified or control PSf/PA membrane. In order to obtain more information of the surface structure of PSf/PA-SiO_2_, an enlarged picture was taken (Figure 1f). It can be observed in Figure 1f that the structure of the showerhead-like bulges are not pores but the sinking of the membrane surface. In other words, the appearance of the membrane sinks or concaves will result in a rougher and higher surface area for the PSf/PA-SiO_2_ membranes. The increase of roughness of PSf/PA-SiO_2_ membranes can be seen clearly from the support information (Figure S1), and the AFM results in supporting information (Figure S2, Table S1) just applied to confirm this change. It can be deduced that the embedding of SiO_2_ into the PA layer would lead to the increase of solvent permeance by providing greater surface area available.

**Figure 2:**
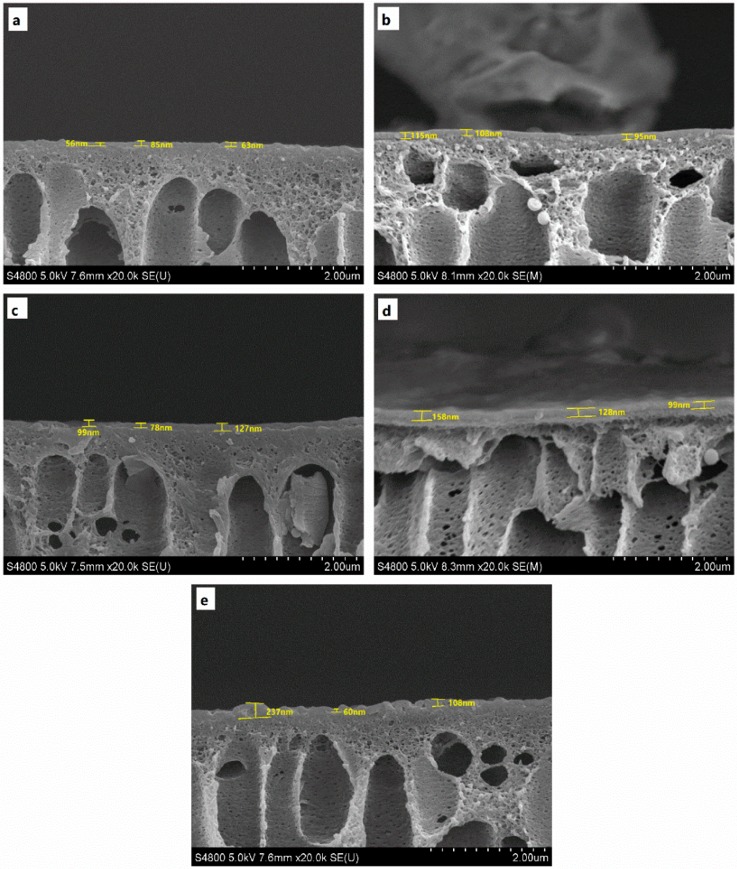
SEM pictures of the cross section for (**a**) PSf/PA and PSf/PA-SiO_2_ composite membrane with the mass fraction of SiO_2_ of (**b**) 0.0125 wt %; (**c**) 0.025 wt %; (**d**) 0.05 wt %; (**e**) 0.075 wt % in the magnification of 20,000 times.

Figure 2 show the smooth area of cross section of the PSf/PA and PSf/PA-SiO_2_ composite membranes, the PSf/PA membrane shows a dense PA layer and the thickness ranges from 56 nm to 85 nm. However, after the introduction of SiO_2_, the PSf/PA-SiO_2_ composite membranes show a more inhomogeneity thickness and are thicker than the controlled PSf/PA membrane, particularly at high concentration of SiO_2_. 

**Figure 3:**
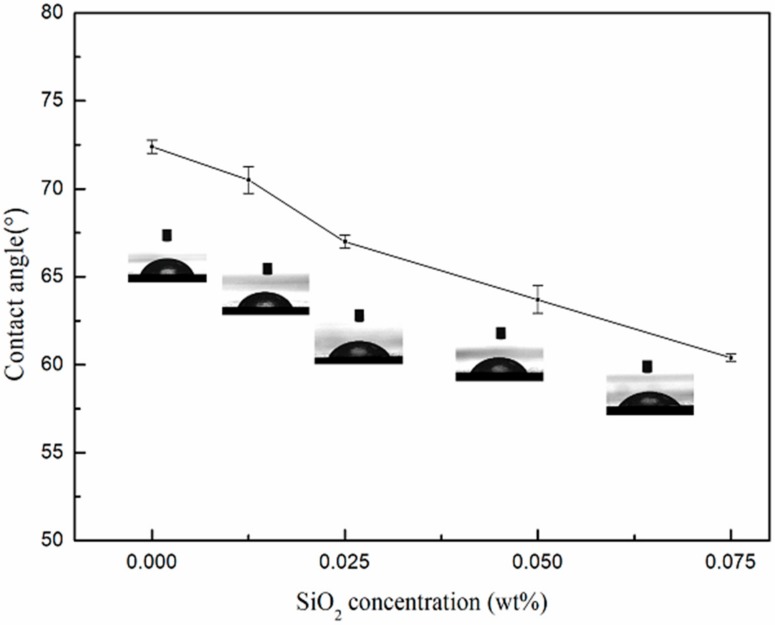
Contact angles of PSf/PA and PSf/PA-SiO_2_ membranes.

The water contact angle of the membranes with different silica concentrations (0 wt %~0.075 wt %) are shown in Figure 3. The contact angle of the pure PSf/PA membrane was 72.4°. After the introduction of silica nanoparticles, the contact angle of the PSf/PA-SiO_2_ membranes decreased from 70.5° to 60.4° with the increase of silica concentration from 0.0125 wt % to 0.075 wt %. The contact angle result indicated that the presence of SiO_2_ in the modified membranes improved the hydrophilicity of the membrane. Two mechanisms were proposed for the improvement, these include, firstly, the abundant –OH functional groups on silica nanoparticles improved the hydrophilicity of silica nanoparticles, which subsequently increased the hydrophilicity of PSf/PA-SiO_2_ membranes. Secondly, the influence of SiO_2_ on the morphology of membrane surfaces is another reason to the decrease of contact angle result when SiO_2_ was introduced into the polyamide layer of membranes. It was observed that the greater the concentration of SiO_2_ in the polyamide layer of membranes, the rougher the membrane surface was, which consequently decreased the contact angles of membranes.

### 3.2. Membrane Performance in Organic Solvent

#### 3.2.1. Influence of SiO_2_ Concentration on Membrane Performance

**Figure 4:**
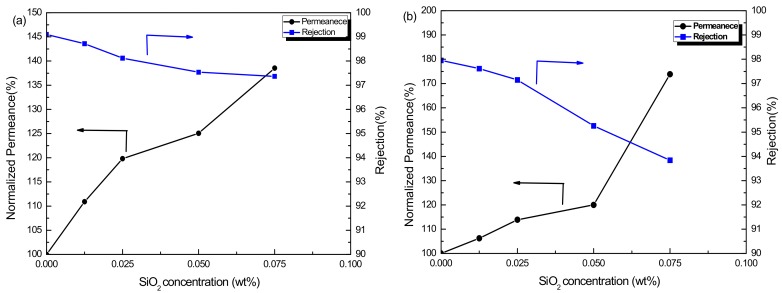
Normalized permeance and rejection of PSf/PA and PSf/PA-SiO_2_ membranes in (**a**) RB/methanol solvent and (**b**) RB/ethanol solvent.

The separation properties of the OSN membranes were investigated via filtration experiments with 20 μM RB methanol and ethanol solutions. The influence of SiO_2_ concentration on membrane performance was shown in Figure 4a,b. The PSf/PA-SiO_2_ composite membranes showed improvement on the filtration performance of both methanol and ethanol solutions. Moreover, the permeance of these membranes increased with the increasing concentration of SiO_2_ nanoparticles. As shown in Figure 4a,b, with the concentration of SiO_2_ increased to 0.075 wt %, the permeance of methanol and ethanol increased by 38.54% and 73.84%, respectively. Meanwhile, the corresponding rejection reduced from 99.1% to 97.4%, and 98.0% to 93.8%, respectively. As a result of larger molecular volume and higher coefficient of viscosity of ethanol compared with methanol, the permeance of ethanol was lower than that of methanol. The improvement of permeance values can be a result of the improvement on hydrophilicity of the PSf/PA-SiO_2_ membranes, which would lead to the acceleration of the penetrating rate of polar alcohol molecules. In addition, as discussed in Section 3.1, the introduction of SiO_2_ increased the roughness of membrane surface, which is capable of increasing the effective area for permeation of alcohol molecules. Furthermore, the introduction of SiO_2_ nanoparticles produced defective areas in the PA layer since they did not participate in the polymerization, which was the major reason for the decrease of RB rejection of the PSf/PA-SiO_2_ membranes. However, the rejection rates of RB in the methanol and ethanol solvents are different from each other based on the same membrane, which perhaps resulted from the adsorption or physical adhesion between dye and solvents.

**Figure 5:**
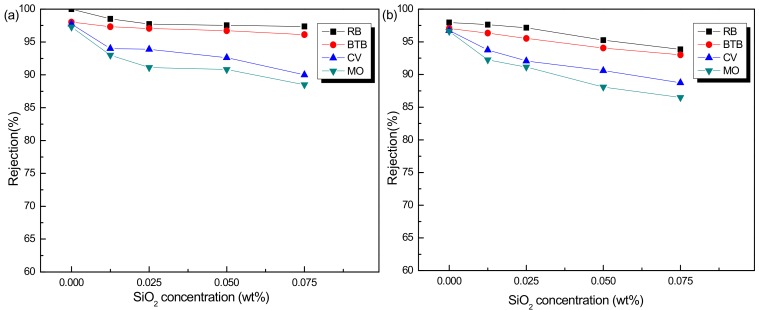
Effect of SiO_2_ concentration on the PSf/PA-SiO_2_ membranes, in terms of rejection for different dyes in (**a**) methanol solvent and (**b**) ethanol solvent.

To further confirm the effect of SiO_2_ concentration on the rejection performance of PSf/PA-SiO_2_ membranes, different dyes with a concentration of 20 μM in methanol and ethanol were employed in the filtration test. The MW of these dyes was shown in Table 2. Figure 5a,b are the rejections of RB, BTB, CV and MO in methanol and ethanol respectively. It can be seen that all the rejection values of these dyes decreased with the increase of SiO_2_ in membranes. Moreover, the reduction degrees of CV and MO were higher than that of RB and BTB. Compared to RB and BTB, CV and MO had lower molecular weights, which was the major reason for larger decreased rejections of CV and MO. The variation in trend of the rejection for these dyes in ethanol solvent (Figure 5b) was similar to the results in methanol solvent which was in the order of RB > BTB > CV > MO. Furthermore, when SiO_2_ concentration was higher than 0.05 wt %, the rejection of PSf/PA-SiO_2_ for MO in methanol and ethanol declined to 88.5% and 86.5% respectively, which indicated that the molecular weight cut-offs of the membranes were more than 327 Da. When SiO_2_ concentration was higher than 0.025 wt % and lower than 0.05 wt %, the rejection of PSf/PA-SiO_2_ for MO in ethanol was lower than 90%. Both the rejection in methanol and ethanol decreased with the increase of SiO_2_ concentration. The above variation can be explained by the defects on the PSf/PA-SiO_2_ membranes. The defective region of the membranes could not provide resistance to the dyes with low molecular weight. Considering the above discussion, we could infer that when SiO_2_ concentration was 0.025 wt %, the PSf/PA-SiO_2_ membrane display relatively excellent performance, high rejection (>90%) of MO, CV, BTB and RB both in methanol and ethanol solvent.

The fabricated PSf/PA-SiO_2_ 0.025% membrane was compared with other thin film nanocomposite membranes reported in the literature. As shown in Table 3, the performance of permeance or rejection in this work is better than some reports, and the reports show both higher permeance and rejection are also difficult to be fabricated with more expensive polymer materials.

#### 3.2.2. Stability Performance of PSf/PA-SiO_2_ Membrane 

**Figure 6:**
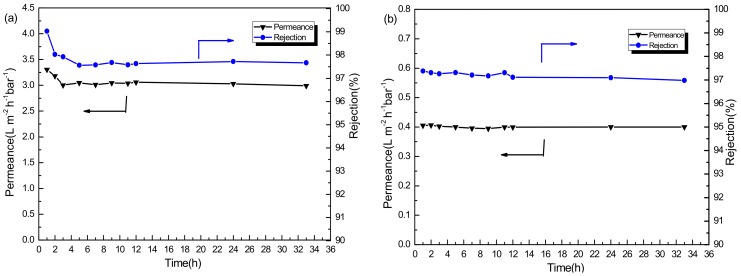
Long-time test of PSf/PA-SiO_2_ 0.025% membrane: (**a**) RB in methanol solution and (**b**) RB in ethanol solution.

To further investigate the stability of the PSf/PA-SiO_2_ membranes, the PSf/PA-SiO_2_ 0.025% membrane with the best performance in the above study was selected and used in long period permeation tests in methanol and ethanol solvent with 20 μM RB respectively. Figure 6 shows the long-term stability of the PSf/PA-SiO_2_ 0.025% membrane in methanol (Figure 6a), and ethanol solvent (Figure 6b). After 33 h, the membrane still showed a stable permeability and retention, confirming the excellent stability of the PSf/PA-SiO_2_ 0.025% membrane. It is interesting that the permeance decrease of methanol was more dramatic compared to ethanol. The major reason behind this is the higher viscosity and larger molecular volume of ethanol. Overall, the prepared PSf/PA-SiO_2_ membrane showed an outstanding separation performance for OSN application.

## 4. Conclusions

PSf/PA-SiO_2_ membranes were firstly fabricated by embedding nanoporous SiO_2_ particles into the PA layer during the IP process, and EDX analysis confirmed the successful addition of SiO_2_. SEM and AFM results indicated an increase of membrane roughness after SiO_2_ introduction. Results from a contact angle test indicated that the hydrophilicity of the membrane was improved after modification with SiO_2_. The performance of the OSN suggested that the permeance of methanol and ethanol for PSf/PA-SiO_2_ membranes increased dramatically with slight decline in rejection. When the SiO_2_ concentration was 0.025 wt %, the PSf/PA-SiO_2_ membrane showed a relatively high rejections of RB, BTB, CV and MO dyes in methanol and ethanol solvents, which were around 90%. The long-term filtration stability test showed the excellent stability property of the newly fabricated PSf/PA-SiO_2_ composite membrane in methanol and ethanol solvents.

## Figures and Tables

**Table 1:** Atomic ratio of different element in the PSf /PA membrane and PSf/PA-SiO_2_ membrane.

Membrane	C	O	S	Cl	N	Si
PSf/PA	72.21	20.41	4.37	0.46	2.55	-
PSf/PA-SiO_2_ 0.075%	69.18	21.30	3.36	0.51	4.77	0.88

**Table 2:** The molecular weight (MW) of different dyes.

Dye	RB	BTB	CV	MO
MW (Da)	1017	624	408	327

**Table 3:** Performance comparison of PSf/PA-SiO_2_ 0.025% membrane with the other TFC membranes reported in the literature.

Membrane Type	Solvent	Permeance (L·m^−2^·h^−1^·bar^−1^)	Marker	Marker MW (g mol^−1^ )	Rejection	Reference
PSf/PA-SiO_2_ 0.025%	Methanol	3.29	RB	1017	98%	This work
			BTB	624	97%	
			CV	408	94%	
			MO	327	91%	
	Ethanol	0.42	RB	1017	97%	
			BTB	624	96%	
			CV	408	92%	
			MO	327	91%	
PA/PSf	Methanol	2	Bromothymol Blue	624	>90%	[34]
PA/crosslinked P84 polyimide	Methanol	1.5	Styrene oligomers	236	98%	[17]
PIM–1/polyacrylonitrile	Methanol	6	Hexaphenylbenzene	535	73%	[39]
	Ethanol	3			78%	
(PIM-1/poly(ethylene imine))/polyacrylonitrile	Methanol	3.6	Hexaphenylbenzene	535	91%	
	Ethanol	1.4			85%	
(PA/MOFs)/P84 polyimide	Methanol	3.9	styrene oligomers	236	96%	[40]
TiO _2_ nanoparticles + PA/ polyimide	Methanol	24	Bromothymol Blue	624	>90%	[41]

## Supplementary Materials

The following are available online at http://www.mdpi.com/2077-0375/8/4/89/s1, Figure S1: SEM pictures of surface morphology of (a) PSf/PA and PSf/PA-SiO2 composite membrane with the mass fraction of SiO2 of (b) 0.0125 wt %; (c) 0.025 wt %; (d) 0.05 wt %; (e) 0.075 wt % in the magnification of 1000 times. Figure S2: AFM 3D images of membrane PSf/PA and PSf/PA-SiO2 membranes. Table S1: Surface roughness parameters of PSf/PA and PSf/PA-SiO2 membranes: Rq, Ra and Rz.
