# Supplementary material for: Polysulfone/Polyamide-SiO2 Composite Membrane with High Permeance for Organic Solvent Nanofiltration
Source: Membranes (Basel). 2018 Oct 3;8(4):89. doi: 10.3390/membranes8040089 (PMC6316106; doi:10.3390/membranes8040089)
Supplement: Supplementary file 1 [file membranes-08-00089-s001.pdf]

membranes

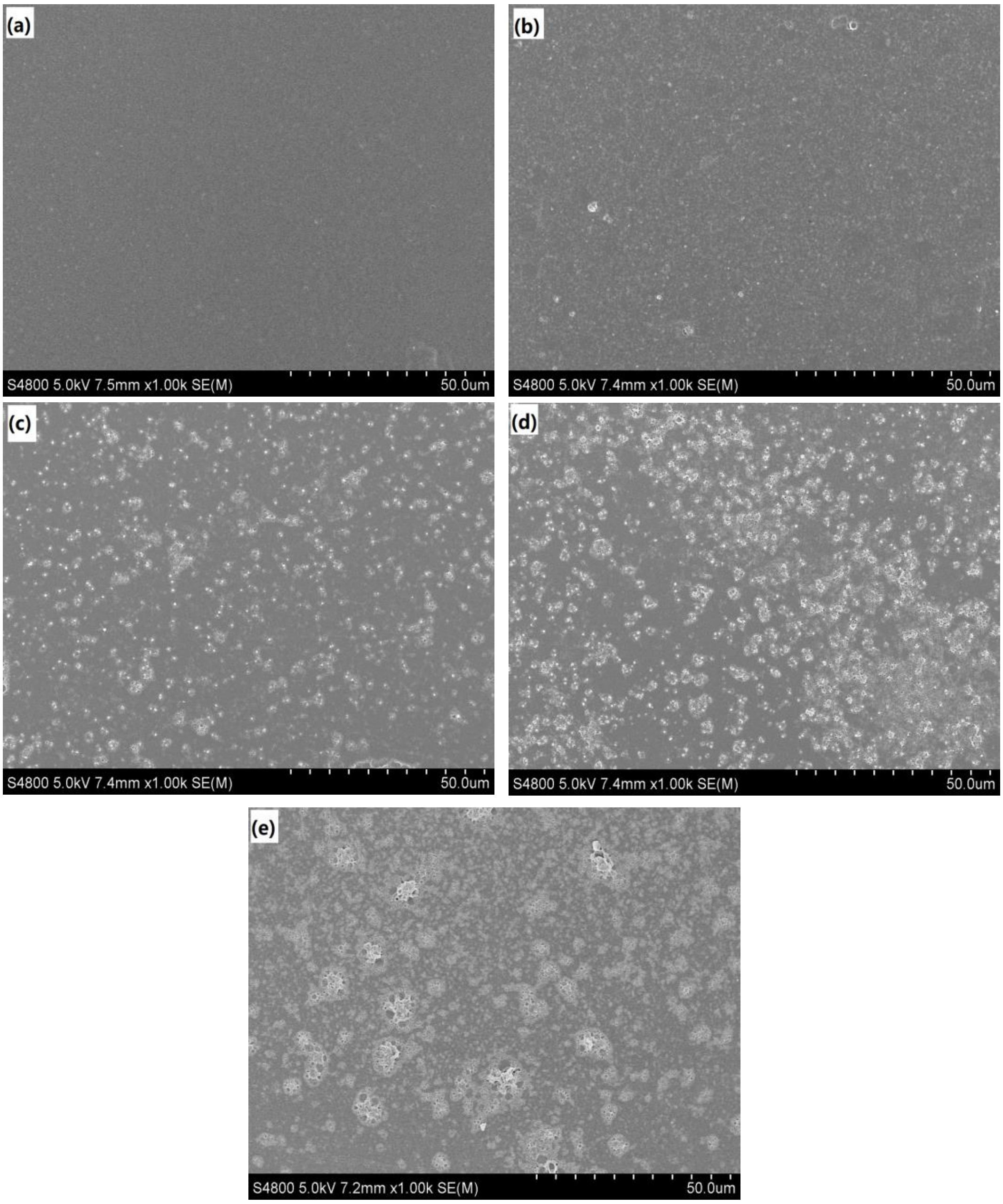


**Figure S1.** SEM pictures of surface morphology of (**a**) PSf/PA and PSf/PA-$SiO_2$ composite membrane with the mass fraction of $SiO_2$ of (**b**) 0.0125 wt %; (**c**) 0.025 wt %; (**d**) 0.05 wt %; (**e**) 0.075 wt % in the magnification of 1000 times.

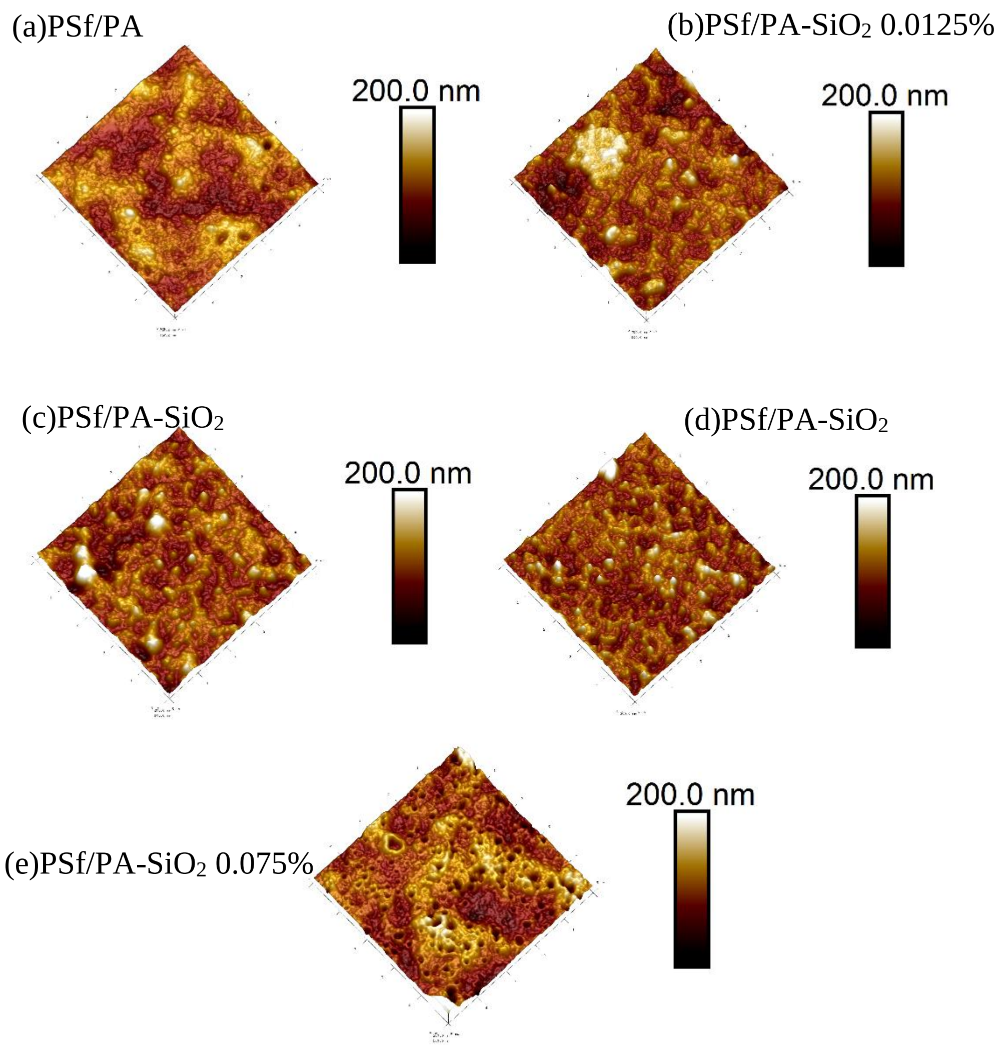


**Figure S2.** AFM 3D images of membrane PSf/PA and PSf/PA-$SiO_2$ membranes.

**Table S1.** Surface roughness parameters of PSf/PA and PSf/PA-$SiO_2$ membranes: Rq, Ra and Rz.

| Membranes | $R_q$ (nm) | $R_a$ (nm) | $R_z$ (nm) |
|---|---|---|---|
| PSf/PA | 18.2 | 12.5 | 170 |
| PSf/PA-$SiO_2$ 0.0125% | 24.2 | 18.5 | 251 |
| PSf/PA-$SiO_2$ 0.025% | 39.4 | 28.1 | 301 |
| PSf/PA-$SiO_2$ 0.05% | 52.6 | 39.3 | 456 |
| PSf/PA-$SiO_2$ 0.075% | 61.6 | 47.3 | 500 |
